# 1,3-Difurfurylbenzimidazolium chloride monohydrate

**DOI:** 10.1107/S1600536809029626

**Published:** 2009-07-29

**Authors:** Mehmet Akkurt, Nihat Şireci, Selma Deniz, Hasan Küçükbay, Orhan Büyükgüngör

**Affiliations:** a Department of Physics, Faculty of Arts and Sciences, Erciyes University, 38039 Kayseri, Turkey; b Department of Chemistry, Faculty of Arts and Sciences, Adıyaman University, 02040 Adıyaman, Turkey; c Department of Chemistry, Faculty of Arts and Sciences, nönü University, 44280 Malatya, Turkey; d Department of Physics, Faculty of Arts & Science, Ondokuz Mayıs University, 55139 Kurupelit-Samsun, Turkey

## Abstract

The title compound, C_17_H_15_N_2_O_2_^+^·Cl^−^·H_2_O, was synthesized from benzimidazole and furfryl chloride in dimethyl­formamide. The cationic benzimidazolium ring is connected to two furan rings *via* methyl­ene bridges. The furan rings make dihedral angle of 79.09 (18)° with respect to each other, and make dihedral angles of 73.92 (12) and 72.58 (13)° with respect to the benzimidazole ring. O—H⋯Cl, C—H⋯O and C—H⋯Cl hydrogen bonds and C—H⋯π inter­actions contribute to the stabilization of the crystal structure. Furthermore, there is a π–π inter­action between adjacent five- and six-membered rings of the benzimidazole groups [centroid–centroid distance = 3.5305 (8) Å].

## Related literature

For the biological activity of furan derivatives, see: Ji *et al.* (2009 ▶). For the anti­microbial activity of a large number of organic and organometallic derivatives of benzimidazole against standard bacterial strains, see: Küçükbay & Durmaz (1997 ▶); Küçükbay *et al.* (2001 ▶, 2004 ▶, 2009 ▶); Çetinkaya *et al.* (1996 ▶). For the catalytic activity of furans, see: Küçükbay *et al.* (1996 ▶). For related structures, see: Yıldırım *et al.* (2007 ▶); Akkurt *et al.* (2006 ▶, 2007 ▶). For bond-length data, see: Allen *et al.* (1987 ▶).
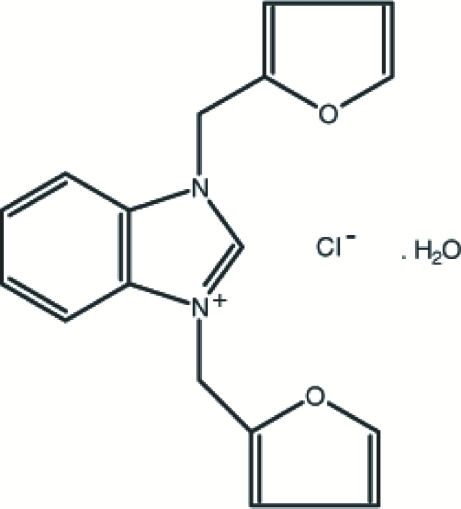


## Experimental

### 

#### Crystal data

C_17_H_15_N_2_O_2_^+^·Cl^−^·H_2_O
*M* *_r_* = 332.78
Triclinic, 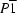

*a* = 9.0201 (5) Å
*b* = 9.3135 (5) Å
*c* = 11.2711 (6) Å
α = 66.778 (4)°
β = 81.869 (4)°
γ = 73.656 (4)°
*V* = 834.50 (8) Å^3^
*Z* = 2
Mo *K*α radiation
μ = 0.25 mm^−1^
*T* = 296 K
0.58 × 0.49 × 0.38 mm

#### Data collection

Stoe IPDS II diffractometer
Absorption correction: integration (*X-RED32*; Stoe & Cie, 2002 ▶) *T*_min_ = 0.871, *T*_max_ = 0.913
15618 measured reflections
3780 independent reflections
2972 reflections with *I* > 2σ(*I*)
*R*_int_ = 0.024

#### Refinement

*R*[*F* ^2^ > 2σ(*F* ^2^)] = 0.041
*wR*(*F* ^2^) = 0.122
*S* = 1.04
3780 reflections
214 parameters
H atoms treated by a mixture of independent and constrained refinement
Δρ_max_ = 0.27 e Å^−3^
Δρ_min_ = −0.21 e Å^−3^

### 

Data collection: *X-AREA* (Stoe & Cie, 2002 ▶); cell refinement: *X-AREA*; data reduction: *X-RED32* (Stoe & Cie, 2002 ▶); program(s) used to solve structure: *SIR97* (Altomare *et al.*, 1999 ▶); program(s) used to refine structure: *SHELXL97* (Sheldrick, 2008 ▶); molecular graphics: *ORTEP-3 for Windows* (Farrugia, 1997 ▶); software used to prepare material for publication: *WinGX* (Farrugia, 1999 ▶) and *PLATON* (Spek, 2009 ▶).

## Supplementary Material

Crystal structure: contains datablocks global, I. DOI: 10.1107/S1600536809029626/xu2569sup1.cif

Structure factors: contains datablocks I. DOI: 10.1107/S1600536809029626/xu2569Isup2.hkl

Additional supplementary materials:  crystallographic information; 3D view; checkCIF report

## Figures and Tables

**Table 1:** Hydrogen-bond geometry (Å, °)

*D*—H⋯*A*	*D*—H	H⋯*A*	*D*⋯*A*	*D*—H⋯*A*
O3—H*W*1⋯Cl1^i^	0.93 (3)	2.27 (3)	3.1563 (17)	159 (3)
O3—H*W*2⋯Cl1	0.98 (3)	2.22 (3)	3.1848 (17)	168 (3)
C7—H7⋯O3	0.93	2.22	3.133 (2)	168
C8—H8*A*⋯Cl1^ii^	0.97	2.75	3.7098 (18)	169
C8—H8*B*⋯Cl1	0.97	2.67	3.6371 (18)	173
C13—H13*A*⋯Cl1^i^	0.97	2.67	3.6332 (18)	171
C13—H13*B*⋯Cl1^iii^	0.97	2.66	3.6290 (19)	177
C11—H11⋯*Cg*2^iv^	0.93	2.85	3.641 (4)	144
C12—H12⋯*Cg*4^iv^	0.93	2.96	3.718 (2)	139

Symmetry codes: (i) 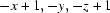; (ii) 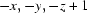; (iii) 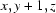; (iv) 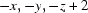. *Cg*2 and *Cg*4 are the centroids of the O2/C14–C17 furan and C1–C6 benzene rings, respectively.

**Fig. 1.**
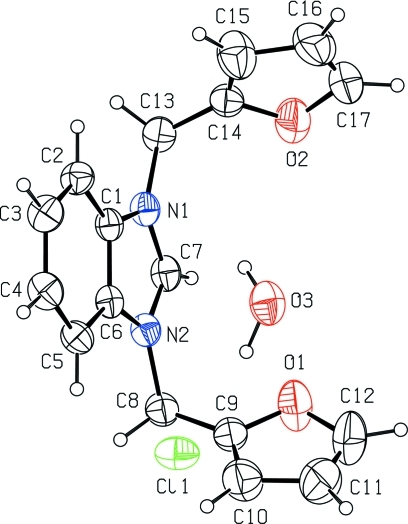
The molecular structure of the title compound, showing the atom-numbering scheme and 30% probability displacement ellipsoids.

**Fig. 2.**
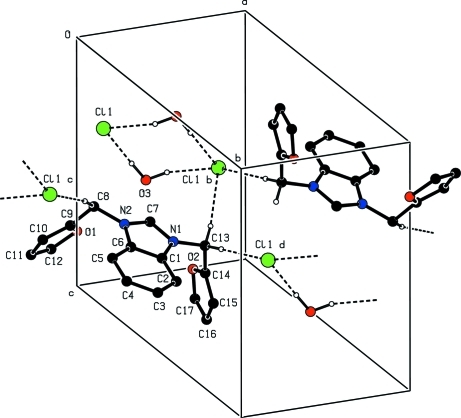
View of the hydrogen bonding of (I) in the unit cell.

## supplementary crystallographic information

### Comment

Like benzimidazoles, furan derivatives occur widely as key structural subunits in numerous natural products, which exhibit interesting biological activities and also in substances of relevance for industry (Ji *et al.*, 2009). Previously, a large number of organic and organometallic derivatives of benzimidazole were prepared in our research laboratory for their antimicrobial activities against standard bacterial strains (Küçükbay & Durmaz, 1997; Küçükbay *et al.*, 2001; Küçükbay *et al.*, 2004; Çetinkaya *et al.*, 1996; Küçükbay *et al.*, 2009) and catalytic activities in some carbon-carbon bond formation reactions and catalytic synthesis of furans (Küçükbay *et al.*, 1996). In connection with these studies, we planned to synthesize having furfuryl substituted new benzimidazole compound (I) and elucidate its crystal structure.

In the asymmetric unit of the title compound (Fig. 1), there are one Cl^-^ anion, a 1,3-di(furfuryl)benzimidazolium cation and one water molecule. The bond lengths are comparable with those found in earlier work on similar compounds (Allen *et al.*, 1987). The O1/C9–C12 and O2/C14–C17 furan rings and N1/N2/C1–C7 benzimidazole ring are almost planar, with maximum deviations of 0.011 (2) for O1 atoms and -0.013 (2) for O2 atom, and -0.008 (1) Å for N1 atom, respectively. The furan rings make dihedral angles of 79.09 (18)° with each other and 73.92 (12) and 72.58 (13)°, respectively, with the benzimidazole ring.

In the crystal structure of (I), there are O—H···Cl, C—H···O and C—H···Cl hydrogen-bonds (Fig. 2) and C—H···π interactions to stabilize the structure (Table 1). Furthermore, there are π–π interactions between the sequential five- and six membered rings {*Cg*2 (ring N1/N2/C1/C6/C7)···*Cg*4 (ring C1–C6) [-*x*, 1 - *x*, 1 - *z*] = 3.5305 (8) Å} of the benzimidazole groups.

### Experimental

A mixture of benzimidazole (1.18 g, 10 mmol) and furfuryl chloride (2.3 g, 20 mmol) in DMF (4 ml) was heated under reflux for 4 h. The solution was allowed to cool to room temperature and Et_2_O (5 ml) was added. The precipitate was then crystallized from EtOH / Et_2_O(2:1). Yield: 1.43 g, 71%, m.p. 488–489 K. ^1^HNMR (DMSO-d_6_): δ 5.93(4*H*, s), 6.51(2*H*, d), 6.84(2*H*, d),8.12(2*H*, d), 7.71 (4*H*, m), 10.21(1*H*, s). ^13^CNMR (DMSO-d_6_): δ 43.05, 111.00, 111.39, 113.99, 126.93, 130.79, 142.37, 144.38, 146.77. Analysis calculated for C_17_H_17_N_2_O_3_Cl: C 61.35, H 5.11, N 8.42%. Found: C 60.97, H 5.06, N 8.38%.

### Refinement

H atoms of the water molecules were located in a difference Fourier map and their positional parameters refined freely, with *U*_iso_(H) = 1.5*U*_eq_(O). The other H atoms were located geometrically and refined using a riding model, with C–H = 0.93 and 0.97 Å, and *U*_iso_(H) = 1.2*U*_eq_(C).

### Crystal data

**Table:**

C_17_H_15_N_2_O_2_^+^.Cl^−^·H_2_O	*Z* = 2
*M**_r_* = 332.78	*F*(000) = 348
Triclinic, *P*1	*D*_x_ = 1.324 Mg m^−^^3^
Hall symbol: -P 1	Mo *K*α radiation, λ = 0.71073 Å
*a* = 9.0201 (5) Å	Cell parameters from 20002 reflections
*b* = 9.3135 (5) Å	θ = 2.0–28.0°
*c* = 11.2711 (6) Å	µ = 0.25 mm^−^^1^
α = 66.778 (4)°	*T* = 296 K
β = 81.869 (4)°	Prism, colourless
γ = 73.656 (4)°	0.58 × 0.49 × 0.38 mm
*V* = 834.50 (8) Å^3^	

### Data collection

**Table:**

Stoe IPDS II diffractometer	3780 independent reflections
Radiation source: sealed X-ray tube, 12 x 0.4 mm long-fine focus	2972 reflections with *I* > 2σ(*I*)
plane graphite	*R*_int_ = 0.024
Detector resolution: 6.67 pixels mm^-1^	θ_max_ = 27.5°, θ_min_ = 2.0°
ω scans	*h* = −11→11
Absorption correction: integration (*X-RED32*; Stoe & Cie, 2002)	*k* = −12→12
*T*_min_ = 0.871, *T*_max_ = 0.913	*l* = −14→14
15618 measured reflections	

### Refinement

**Table:**

Refinement on *F*^2^	Primary atom site location: structure-invariant direct methods
Least-squares matrix: full	Secondary atom site location: difference Fourier map
*R*[*F*^2^ > 2σ(*F*^2^)] = 0.041	Hydrogen site location: inferred from neighbouring sites
*wR*(*F*^2^) = 0.122	H atoms treated by a mixture of independent and constrained refinement
*S* = 1.04	*w* = 1/[σ^2^(*F*_o_^2^) + (0.0718*P*)^2^ + 0.0531*P*] where *P* = (*F*_o_^2^ + 2*F*_c_^2^)/3
3780 reflections	(Δ/σ)_max_ < 0.001
214 parameters	Δρ_max_ = 0.27 e Å^−^^3^
0 restraints	Δρ_min_ = −0.21 e Å^−^^3^

### Special details

**Table:**

Geometry. Bond distances, angles *etc*. have been calculated using the rounded fractional coordinates. All su's are estimated from the variances of the (full) variance-covariance matrix. The cell e.s.d.'s are taken into account in the estimation of distances, angles and torsion angles
Refinement. Refinement on *F*^2^ for ALL reflections except those flagged by the user for potential systematic errors. Weighted *R*-factors *wR* and all goodnesses of fit *S* are based on *F*^2^, conventional *R*-factors *R* are based on *F*, with *F* set to zero for negative *F*^2^. The observed criterion of *F*^2^ > σ(*F*^2^) is used only for calculating -*R*-factor-obs *etc*. and is not relevant to the choice of reflections for refinement. *R*-factors based on *F*^2^ are statistically about twice as large as those based on *F*, and *R*-factors based on ALL data will be even larger.

### Fractional atomic coordinates and isotropic or equivalent isotropic displacement parameters (Å^2^)

**Table:**

	*x*	*y*	*z*	*U*_iso_*/*U*_eq_	
O1	0.13050 (17)	−0.1335 (2)	0.86538 (14)	0.1035 (5)	
O2	0.4854 (2)	0.20834 (19)	0.87489 (17)	0.1120 (7)	
N1	0.23659 (12)	0.34379 (14)	0.66557 (10)	0.0532 (3)	
N2	0.09145 (13)	0.19641 (13)	0.65802 (11)	0.0538 (3)	
C1	0.08420 (14)	0.43162 (16)	0.66979 (12)	0.0494 (4)	
C2	0.02240 (17)	0.58200 (18)	0.67695 (15)	0.0603 (4)	
C3	−0.13677 (18)	0.63291 (19)	0.67798 (17)	0.0681 (5)	
C4	−0.22951 (17)	0.5392 (2)	0.67213 (16)	0.0676 (5)	
C5	−0.16859 (16)	0.38960 (19)	0.66484 (14)	0.0590 (4)	
C6	−0.00818 (15)	0.33787 (16)	0.66462 (12)	0.0497 (4)	
C7	0.23474 (16)	0.20505 (17)	0.65930 (13)	0.0558 (4)	
C8	0.04576 (19)	0.05737 (18)	0.65596 (15)	0.0624 (5)	
C9	0.0118 (2)	−0.05143 (19)	0.78656 (15)	0.0644 (5)	
C10	−0.1182 (3)	−0.0852 (3)	0.8477 (2)	0.0975 (8)	
C11	−0.0776 (4)	−0.1983 (4)	0.9725 (3)	0.1193 (11)	
C12	0.0683 (4)	−0.2261 (3)	0.9797 (2)	0.1176 (10)	
C13	0.37621 (16)	0.3936 (2)	0.67077 (15)	0.0617 (4)	
C14	0.41286 (17)	0.3570 (2)	0.80414 (16)	0.0650 (5)	
C15	0.3759 (3)	0.4445 (3)	0.8759 (2)	0.1043 (9)	
C16	0.4334 (4)	0.3444 (4)	1.0001 (2)	0.1183 (13)	
C17	0.4990 (4)	0.2065 (4)	0.9964 (2)	0.1177 (11)	
O3	0.50690 (18)	−0.09917 (19)	0.68142 (14)	0.0861 (5)	
Cl1	0.33115 (5)	−0.17640 (6)	0.49633 (5)	0.0794 (2)	
H2	0.08430	0.64470	0.68080	0.0720*	
H3	−0.18370	0.73320	0.68270	0.0820*	
H4	−0.33630	0.57900	0.67320	0.0810*	
H5	−0.23060	0.32740	0.66040	0.0710*	
H7	0.32240	0.12480	0.65620	0.0670*	
H8A	−0.04510	0.09600	0.60450	0.0750*	
H8B	0.12860	−0.00190	0.61540	0.0750*	
H10	−0.21680	−0.04260	0.81460	0.1170*	
H11	−0.14480	−0.24430	1.03780	0.1430*	
H12	0.12490	−0.29850	1.05160	0.1410*	
H13A	0.46300	0.33850	0.63050	0.0740*	
H13B	0.36030	0.50870	0.62220	0.0740*	
H15	0.32200	0.55240	0.84990	0.1250*	
H16	0.42470	0.37400	1.07100	0.1420*	
H17	0.54860	0.11780	1.06480	0.1410*	
HW1	0.575 (3)	−0.041 (3)	0.627 (3)	0.1190*	
HW2	0.461 (3)	−0.139 (3)	0.631 (3)	0.1190*	

### Atomic displacement parameters (Å^2^)

**Table:**

	*U*^11^	*U*^22^	*U*^33^	*U*^12^	*U*^13^	*U*^23^
O1	0.0817 (9)	0.1176 (11)	0.0752 (8)	−0.0088 (8)	−0.0101 (7)	−0.0070 (8)
O2	0.1417 (15)	0.0792 (9)	0.1012 (11)	−0.0119 (9)	−0.0444 (10)	−0.0164 (8)
N1	0.0444 (5)	0.0560 (6)	0.0524 (6)	−0.0049 (4)	−0.0014 (4)	−0.0183 (5)
N2	0.0547 (6)	0.0514 (6)	0.0531 (6)	−0.0036 (5)	−0.0051 (4)	−0.0227 (5)
C1	0.0449 (6)	0.0519 (7)	0.0450 (6)	−0.0053 (5)	−0.0021 (5)	−0.0159 (5)
C2	0.0582 (8)	0.0536 (7)	0.0680 (8)	−0.0090 (6)	−0.0023 (6)	−0.0250 (6)
C3	0.0610 (8)	0.0584 (8)	0.0812 (10)	0.0015 (7)	−0.0019 (7)	−0.0335 (7)
C4	0.0483 (7)	0.0708 (10)	0.0785 (10)	0.0008 (7)	−0.0030 (7)	−0.0330 (8)
C5	0.0484 (7)	0.0665 (8)	0.0624 (8)	−0.0092 (6)	−0.0047 (6)	−0.0268 (7)
C6	0.0499 (6)	0.0504 (7)	0.0443 (6)	−0.0042 (5)	−0.0039 (5)	−0.0178 (5)
C7	0.0515 (7)	0.0553 (7)	0.0529 (7)	0.0004 (5)	−0.0030 (5)	−0.0212 (6)
C8	0.0729 (9)	0.0566 (8)	0.0621 (8)	−0.0081 (6)	−0.0080 (7)	−0.0302 (6)
C9	0.0726 (9)	0.0580 (8)	0.0649 (8)	−0.0120 (7)	−0.0069 (7)	−0.0267 (7)
C10	0.0818 (13)	0.1172 (17)	0.0885 (13)	−0.0348 (12)	−0.0027 (10)	−0.0263 (12)
C11	0.124 (2)	0.137 (2)	0.0860 (15)	−0.0649 (18)	0.0070 (14)	−0.0115 (14)
C12	0.130 (2)	0.1138 (18)	0.0697 (13)	−0.0187 (16)	−0.0097 (13)	0.0007 (12)
C13	0.0451 (6)	0.0682 (9)	0.0636 (8)	−0.0135 (6)	0.0039 (6)	−0.0184 (7)
C14	0.0531 (7)	0.0719 (9)	0.0681 (9)	−0.0224 (7)	−0.0034 (6)	−0.0190 (7)
C15	0.1222 (18)	0.1028 (16)	0.0819 (13)	0.0002 (13)	−0.0143 (12)	−0.0438 (12)
C16	0.150 (2)	0.147 (3)	0.0756 (13)	−0.062 (2)	−0.0139 (14)	−0.0395 (15)
C17	0.147 (2)	0.114 (2)	0.0847 (15)	−0.0519 (18)	−0.0473 (15)	−0.0017 (14)
O3	0.0829 (8)	0.0841 (9)	0.0778 (8)	−0.0003 (6)	−0.0117 (6)	−0.0271 (7)
Cl1	0.0681 (3)	0.0830 (3)	0.1058 (4)	−0.0237 (2)	0.0067 (2)	−0.0545 (3)

### Geometric parameters (Å, °)

**Table:**

O1—C9	1.339 (2)	C11—C12	1.276 (5)
O1—C12	1.382 (3)	C13—C14	1.469 (2)
O2—C14	1.322 (3)	C14—C15	1.312 (3)
O2—C17	1.385 (3)	C15—C16	1.415 (3)
O3—HW1	0.93 (3)	C16—C17	1.268 (5)
O3—HW2	0.98 (3)	C2—H2	0.9300
N1—C1	1.3929 (19)	C3—H3	0.9300
N1—C7	1.326 (2)	C4—H4	0.9300
N1—C13	1.475 (2)	C5—H5	0.9300
N2—C6	1.393 (2)	C7—H7	0.9300
N2—C8	1.475 (2)	C8—H8A	0.9700
N2—C7	1.320 (2)	C8—H8B	0.9700
C1—C2	1.384 (2)	C10—H10	0.9300
C1—C6	1.387 (2)	C11—H11	0.9300
C2—C3	1.379 (2)	C12—H12	0.9300
C3—C4	1.392 (2)	C13—H13B	0.9700
C4—C5	1.377 (3)	C13—H13A	0.9700
C5—C6	1.390 (2)	C15—H15	0.9300
C8—C9	1.469 (2)	C16—H16	0.9300
C9—C10	1.326 (3)	C17—H17	0.9300
C10—C11	1.408 (4)		
			
Cl1···C8	3.6371 (18)	C5···H8A	2.9500
Cl1···C13^i^	3.6290 (19)	C8···H5	3.0100
Cl1···O3^ii^	3.1563 (17)	C13···H2	3.0100
Cl1···O3	3.1848 (17)	C14···H11^ix^	2.9800
Cl1···C13^ii^	3.6332 (18)	C15···H16^viii^	3.0400
Cl1···H13A^ii^	2.6700	C15···H11^ix^	2.9900
Cl1···H8B	2.6700	C16···H16^viii^	3.0200
Cl1···H13B^i^	2.6600	HW1···Cl1^ii^	2.27 (3)
Cl1···H8A^iii^	2.7500	HW1···H3^v^	2.5100
Cl1···HW2	2.22 (3)	HW1···H7	2.4300
Cl1···H5^iii^	3.0100	H2···C13	3.0100
Cl1···HW1^ii^	2.27 (3)	H2···H13B	2.5800
O1···N2	2.998 (2)	HW2···Cl1	2.22 (3)
O1···C7	3.391 (2)	HW2···H7	2.5300
O2···N1	3.123 (2)	H3···O3^x^	2.7900
O3···C17^iv^	3.362 (3)	H3···HW1^x^	2.5100
O3···Cl1	3.1848 (17)	H5···H8A	2.5700
O3···C7	3.133 (2)	H5···Cl1^iii^	3.0100
O3···Cl1^ii^	3.1563 (17)	H5···C8	3.0100
O3···H3^v^	2.7900	H7···O3	2.2200
O3···H7	2.2200	H7···HW1	2.4300
O3···H17^iv^	2.7800	H7···H8B	2.5400
N1···N2	2.1772 (17)	H7···H13A	2.5500
N1···O2	3.123 (2)	H7···HW2	2.5300
N2···O1	2.998 (2)	H8A···C5	2.9500
N2···N1	2.1772 (17)	H8A···H5	2.5700
C1···C6^vi^	3.5787 (18)	H8A···Cl1^iii^	2.7500
C1···C5^vi^	3.5392 (19)	H8B···Cl1	2.6700
C4···C7^vi^	3.550 (2)	H8B···H7	2.5400
C5···C1^vi^	3.5392 (19)	H11···C14^ix^	2.9800
C6···C1^vi^	3.5787 (18)	H11···C15^ix^	2.9900
C7···O3	3.133 (2)	H12···C5^ix^	3.0200
C7···O1	3.391 (2)	H13A···H7	2.5500
C7···C4^vi^	3.550 (2)	H13A···Cl1^ii^	2.6700
C8···Cl1	3.6371 (18)	H13B···C2	2.9600
C13···Cl1^ii^	3.6332 (18)	H13B···H2	2.5800
C13···Cl1^vii^	3.6290 (19)	H13B···Cl1^vii^	2.6600
C16···C16^viii^	3.435 (5)	H16···C15^viii^	3.0400
C17···O3^iv^	3.362 (3)	H16···C16^viii^	3.0200
C2···H13B	2.9600	H17···O3^iv^	2.7800
C5···H12^ix^	3.0200		
			
C9—O1—C12	105.62 (19)	O2—C17—C16	109.6 (2)
C14—O2—C17	106.9 (2)	C1—C2—H2	122.00
HW1—O3—HW2	108 (3)	C3—C2—H2	122.00
C1—N1—C13	126.21 (13)	C4—C3—H3	119.00
C7—N1—C13	125.65 (13)	C2—C3—H3	119.00
C1—N1—C7	108.13 (12)	C5—C4—H4	119.00
C6—N2—C8	126.23 (13)	C3—C4—H4	119.00
C7—N2—C8	125.52 (13)	C4—C5—H5	122.00
C6—N2—C7	108.19 (13)	C6—C5—H5	122.00
N1—C1—C2	131.54 (13)	N1—C7—H7	125.00
C2—C1—C6	122.08 (13)	N2—C7—H7	125.00
N1—C1—C6	106.38 (13)	N2—C8—H8B	109.00
C1—C2—C3	115.74 (15)	C9—C8—H8A	109.00
C2—C3—C4	122.20 (17)	C9—C8—H8B	109.00
C3—C4—C5	122.28 (16)	H8A—C8—H8B	108.00
C4—C5—C6	115.51 (15)	N2—C8—H8A	109.00
N2—C6—C1	106.58 (12)	C9—C10—H10	127.00
C1—C6—C5	122.18 (14)	C11—C10—H10	127.00
N2—C6—C5	131.24 (14)	C12—C11—H11	126.00
N1—C7—N2	110.72 (13)	C10—C11—H11	126.00
N2—C8—C9	111.85 (13)	C11—C12—H12	125.00
O1—C9—C10	109.98 (16)	O1—C12—H12	125.00
C8—C9—C10	132.75 (18)	N1—C13—H13A	109.00
O1—C9—C8	117.25 (16)	C14—C13—H13A	109.00
C9—C10—C11	106.4 (2)	C14—C13—H13B	109.00
C10—C11—C12	107.5 (3)	N1—C13—H13B	109.00
O1—C12—C11	110.4 (2)	H13A—C13—H13B	108.00
N1—C13—C14	111.84 (13)	C16—C15—H15	126.00
O2—C14—C15	109.18 (18)	C14—C15—H15	126.00
C13—C14—C15	131.51 (19)	C15—C16—H16	127.00
O2—C14—C13	119.08 (17)	C17—C16—H16	126.00
C14—C15—C16	107.3 (2)	O2—C17—H17	125.00
C15—C16—C17	107.0 (2)	C16—C17—H17	125.00
			
C12—O1—C9—C10	1.9 (3)	N1—C1—C6—N2	0.25 (14)
C9—O1—C12—C11	−2.1 (3)	N1—C1—C6—C5	−178.98 (12)
C12—O1—C9—C8	−179.56 (18)	N1—C1—C2—C3	179.33 (14)
C17—O2—C14—C15	−2.5 (3)	C2—C1—C6—C5	0.7 (2)
C17—O2—C14—C13	−177.5 (2)	C6—C1—C2—C3	−0.3 (2)
C14—O2—C17—C16	2.3 (4)	C1—C2—C3—C4	0.0 (2)
C13—N1—C1—C6	−178.92 (12)	C2—C3—C4—C5	−0.1 (3)
C7—N1—C13—C14	−95.10 (18)	C3—C4—C5—C6	0.4 (2)
C13—N1—C1—C2	1.4 (2)	C4—C5—C6—N2	−179.78 (14)
C13—N1—C7—N2	178.99 (12)	C4—C5—C6—C1	−0.8 (2)
C1—N1—C13—C14	83.09 (18)	N2—C8—C9—C10	110.2 (3)
C7—N1—C1—C6	−0.47 (14)	N2—C8—C9—O1	−67.9 (2)
C1—N1—C7—N2	0.53 (15)	C8—C9—C10—C11	−179.3 (2)
C7—N1—C1—C2	179.86 (15)	O1—C9—C10—C11	−1.1 (3)
C7—N2—C8—C9	94.03 (18)	C9—C10—C11—C12	−0.2 (4)
C7—N2—C6—C1	0.06 (15)	C10—C11—C12—O1	1.4 (4)
C7—N2—C6—C5	179.19 (14)	N1—C13—C14—O2	80.2 (2)
C6—N2—C7—N1	−0.37 (15)	N1—C13—C14—C15	−93.5 (3)
C8—N2—C6—C1	177.49 (12)	O2—C14—C15—C16	1.7 (3)
C6—N2—C8—C9	−82.98 (18)	C13—C14—C15—C16	176.0 (2)
C8—N2—C7—N1	−177.83 (12)	C14—C15—C16—C17	−0.3 (4)
C8—N2—C6—C5	−3.4 (2)	C15—C16—C17—O2	−1.2 (4)
C2—C1—C6—N2	179.96 (13)		

Symmetry codes: (i) *x*, *y*−1, *z*; (ii) −*x*+1, −*y*, −*z*+1; (iii) −*x*, −*y*, −*z*+1; (iv) −*x*+1, −*y*, −*z*+2; (v) *x*+1, *y*−1, *z*; (vi) −*x*, −*y*+1, −*z*+1; (vii) *x*, *y*+1, *z*; (viii) −*x*+1, −*y*+1, −*z*+2; (ix) −*x*, −*y*, −*z*+2; (x) *x*−1, *y*+1, *z*.

### Hydrogen-bond geometry (Å, °)

**Table:**

*D*—H···*A*	*D*—H	H···*A*	*D*···*A*	*D*—H···*A*
O3—HW1···Cl1^ii^	0.93 (3)	2.27 (3)	3.1563 (17)	159 (3)
O3—HW2···Cl1	0.98 (3)	2.22 (3)	3.1848 (17)	168 (3)
C7—H7···O3	0.93	2.22	3.133 (2)	168
C8—H8A···Cl1^iii^	0.97	2.75	3.7098 (18)	169
C8—H8B···Cl1	0.97	2.67	3.6371 (18)	173
C13—H13A···Cl1^ii^	0.97	2.67	3.6332 (18)	171
C13—H13B···Cl1^vii^	0.97	2.66	3.6290 (19)	177
C11—H11···Cg2^ix^	0.93	2.85	3.641 (4)	144
C12—H12···Cg4^ix^	0.93	2.96	3.718 (2)	139

Symmetry codes: (ii) −*x*+1, −*y*, −*z*+1; (iii) −*x*, −*y*, −*z*+1; (vii) *x*, *y*+1, *z*; (ix) −*x*, −*y*, −*z*+2.
